# Identification of genomic regions associated with shoot fly resistance in maize and their syntenic relationships in the sorghum genome

**DOI:** 10.1371/journal.pone.0234335

**Published:** 2020-06-09

**Authors:** Yogesh Vikal, Arshpreet Kaur, Jawala Jindal, Kirandeep Kaur, Dharminder Pathak, Tosh Garg, Ashanpreet Singh, Princepal Singh, Inderjit Yadav

**Affiliations:** 1 School of Agricultural Biotechnology, Punjab Agricultural University, Ludhiana, India; 2 Department of Plant Breeding and Genetics, Punjab Agricultural University, Ludhiana, India; USDA Agricultural Research Service, UNITED STATES

## Abstract

Shoot fly (*Atherigona naqvii*) is one of the major insects affecting spring maize in North India and can cause yield loss up to 60 per cent. The genetics of insect resistance is complex as influenced by genotypic background, insect population and climatic conditions. Therefore, quantitative trait loci (QTL) mapping is a highly effective approach for studying genetically complex forms of insect resistance. The objective of the present study was to dissect the genetic basis of resistance and identification of genomic regions associated with shoot fly resistance. A total of 107 F_2_ population derived from the cross CM143 (resistant) x CM144 (susceptible) was genotyped with 120 SSR markers. Phenotypic data were recorded on replicated F_2:3_ progenies for various component traits imparting resistance to shoot fly at different time intervals. Resistance to shoot fly was observed to be under polygenic control as evidenced by the identification of 19 putative QTLs governed by overdominance to partial dominance and additive gene actions. The major QTLs conditioning shoot fly resistance viz., *qDH9*.*1* (deadheart) and *qEC9*.*1* (oviposition) explaining 15.03 and 18.89 per cent phenotypic variance, respectively were colocalized on chromosome 9. These QTLs are syntenic to regions of chromosome 10 of sorghum which were also accounted for deadheart and oviposition suggesting that the same gene block may be responsible for shoot fly resistance. The candidate genes such as *cysteine protease*, *subtilisin-chymotrypsin inhibitor*, cytochrome P450 involved in synthesis of alleochemicals, receptor kinases, *glossy15* and ubiquitin-proteasome degradation pathway were identified within the predicted QTL regions. This is the first reported mapping of QTLs conferring resistance to shoot fly in maize, and the markers identified here will be a valuable resource for developing elite maize cultivars with resistance to shoot fly.

## 1. Introduction

Globally maize is the third most important cereal crop after wheat and rice in terms of area and production having diversified uses as food, feed and a range of industrial products. In India, it was cultivated on an area of 9.63 million hectares with annual production of 25.90 million metric tonnes and average productivity of 2.69 metric tonnes per hectare during 2018 (www.indiastat.com). However, maize production is limited by insect pests [1] at different crop growth stages, thus hampering with the realization of yield potential. The continuous planting of maize throughout the year has led to increased incidence of shoot fly (*Atherigona* species) at seedling stage [2]. Sixteen shoot fly species have been reported on maize in Africa and Asia [3], of which *A*. *naqvii* Steyskal (Muscidae: Diptera) is most prevalent in North India [4] and reported to cause a loss of about 28–45 per cent in grain yield during spring season in the Indian Punjab [2].

The *A*. *naqvii* adult female lays eggs singly or in small groups on the stem above the ground or on/in cracks and crevices around the plants in the soil and on the under surface of the cotyledonary or first leaf of young seedlings. The maggots of shoot fly attack the whorl leaves of emerging seedlings causing deadheart while curled and distorted leaves are formed in bigger plants. The soil application of carbofuran 3 G @ 12.5 kg and phorate 10 G @ 10.0 kg per hectare at sowing time [5] or seed treatment with imidacloprid 600 FS @ 6 ml per kg seed one day before sowing has been found effective and recommended for management of shoot fly [6]. However, the intensive usage of insecticides leads to environmental pollution, kills natural enemies of the target pest, may also result in development of insecticide resistance in shoot fly populations. Besides, shoot fly is not easily exposed to insecticides in maize as the larvae feed inside the leaf whorls.

Genetic resistance is the most viable and sustainable strategy for shoot fly management. Low to moderate levels of resistance have been identified against shoot fly in the maize germplasm [7]. However, the genetics of shoot fly resistance in maize has not been investigated in details and no known source of cultivated maize accession is reported to confer absolute resistance to shoot fly. Plant resistance to *Atherigona* spp. is a complex trait and it depends on the interplay of several component characters [2]. Studies conducted in sorghum revealed that resistance to shoot fly was quantitative in nature [8, 9], with predominantly additive gene effects [10]. A series of past studies indicated that the resistance to different insect pests in maize is polygenic in nature. Various types of gene actions such as additive, dominant, and non-additive along with significant genotype by environment interactions have been reported in maize for resistance to storage pests [11] and stem borer [12, 13].

Substantial yield reduction caused by shoot fly incidence in maize has prompted maize workers to accelerate breeding research on shoot fly resistance in India. Conventional breeding for resistance to insect pest is a challenge due to complex inheritance [14]. The availability of molecular markers and development of linkage maps have led to the identification of genomic regions harbouring quantitative trait loci (QTL) for the traits of interest. Once marker-trait association is established, marker-assisted selection (MAS) could be followed to transfer resistance gene/QTLs in elite susceptible backgrounds. To date, several QTL mapping studies are available for storage pest species such as maize weevil [11, 15] and stem borer species such as European corn borer [16, 17], sugarcane borer [18], Southwestern corn borer [19, 20], Mediterranean corn borer [21] in maize. To the best of our knowledge, no report on the molecular mapping of QTL conferring shoot fly resistance in maize is available. Therefore, a detailed study of the underlying genetic basis of resistance to shoot fly in maize is central for designing more effective breeding strategies. Thus, the present study aims (i) to study the genetics and mapping of resistance to shoot fly in maize (ii) to compare the previously published genomic regions involved in different maize insect-pest resistance (iii) to identify syntenic regions associated with resistance to shoot fly in maize and sorghum and (iv) to understand the mechanism of shoot fly resistance.

We present here the first report on mapping QTLs conferring resistance to shoot fly in maize. A number of potential candidate genes were identified spanning within QTL regions which were involved directly or indirectly in shoot fly resistance. Syntenic regions for shoot fly resistance between maize and sorghum were found. Also, it was observed that some of the detected genomic regions were common for various insect-pests resistance in maize.

## 2. Materials and methods

### 2.1 Plant material

The experimental plant material consisted of two parental inbred lines *viz*. CM143 and CM144 and 107 F_2_ individuals & their F_2:3_ families. CM143 moderately resistant to shoot fly, is a well-adapted inbred line, whereas CM144 is susceptible to shoot fly. CM143 and CM144 are the parental lines of a popular hybrid, JH3459 recommended for cultivation in North-Western plains of India. The 107 F_2:3_ families along with parents (CM143 and CM144) were raised in randomized complete block design in two replications at Punjab Agricultural University, Ludhiana (S1 Fig). Each F_2:3_ family was planted in one row of three-meter length accommodating 16 plants with a plant to plant and row to row distance of 20 cm and 60 cm, respectively. Standard agronomical practices were followed for raising the crop. The F_2:3_ families were self-fertilized to generate F_3:4_ families.

### 2.2 Phenotypic evaluation

The fish-meal technique was used for increasing the shoot fly pressure under field conditions [7]. The moistened fish meal was applied @50 g/m^2^ one day after the seedling emergence by broadcasting to screen F_2:3_ families against shoot fly. The data on shoot fly infestation were recorded from ten plants of each F_2:3_ family at different time intervals. Phenotypic data on various traits such as egg count (oviposition, EC), leaf injury (LI), deadheart (DH), leaf glossiness (LG), seedling vigor (SV), leaf sheath pigmentation (LSP), leaf surface wetness (LSW), leaf length (LL), leaf width (LW), leaf area (LA), and stem girth (SG) were recorded. Ovipositional count as total number of eggs on ten random plants of each F_2:3_ family was recorded (S2 Fig). The mean number of eggs per family was calculated on 5, 10, and 15 days after emergence (DAE). The mean values of LI and DH per cent were calculated as number of plants with leaf injury or dead hearts / total number of plants × 100 at 7, 14, and 21 DAE. Data on LI and DH were recorded at different time intervals as these are expressed at early seedling stage to V5-V6 stage (21 days of germination). The percentages of the LI and DH were converted into different scales of 1–5 and 1–7, respectively as given in Table 1. The LI is the initial symptom and DH are formed later. However, the extent of DH formation depends on the host genotype irrespective of initial LI. Hence, different scales were used for these traits. The scale used was modified from Sharma *et al* [22]. The mean value of EC at 15 DAE was pursued for QTL analysis. The average values of LI and DH per cent recorded on 21 DAE were used for QTL identification. Leaf glossiness was visually scored on a scale of 1–5 at 5-leaf stage when there was maximum reflection of light from the leaf surfaces in the early morning hours [23]. The ranking of SV, LSP, and LSW was rated at 5-leaf stage on a 1–5 scale (S2 Fig) and was used to classify the families into different categories (Table 1). The LL (cm), LW (cm), LA (cm^2^), and SG (cm) was also recorded from seedlings at 5-leaf stage. Leaf width was recorded from upper, middle and lower portion of each leaf with measuring scale and its mean was calculated. The observations for LSP, SV, LG, LSW and leaf dimensions were recorded only once at the appropriate stage for influencing shoot fly oviposition and damage behaviour.

**Table 1:** Scale used to record the data on different parameters from CM143, CM144 and 107 F_2:3_ families derived from the cross of CM143 × CM144.

Scale^a^	% Leaf Injury (LI)	% Deadheart (DH)	Leaf surface wetness^b^ (LSW)	Seedling vigour (SV)	Leaf glossiness^c^ (LG)	Leaf sheath pigmentation (LSP)
1 (R)^d^	0–15	0–5	Entire leaf blade densely covered with water droplets	Highly vigorous (plants showing maximum height, more number of fully expanded leaves, good adaptation and robust seedlings)	Highly glossy (light green, shining, narrow and erect leaves)	Leaf sheath with dark pink pigment
2 (MR)	15.1–25	5.1–10	Water droplets spread all over the leaf blade	Vigorous (good plant height, good number of fully expanded leaves, good adaptation and seedling growth)	Glossy (light green, less shining, narrow and erect leaves)	Leaf sheath with fair pink pigment
3 (MR)	25.1–35	10.1–15	Leaf blade near mid rib covered with water droplets	Moderately vigorous (moderate plant height with moderate number of fully expanded leaves and fairly good seedling growth)	Moderate glossy (fair green, light shining, medium leaf width and less drooping leaves)	Leaf sheath with light pink pigment
4 (MS)	35.1–45	15.1–20	Leaf blade with sparsely placed few water droplets	Less vigorous (less plant height with poor leaf expansion and poor adaptation)	Moderate nonglossy (green, pseudo-shine, broad and drooping leaves)	Leaf sheath with very light pink pigment
5 (S)	45.1–55	20.1–25	Leaf blade without water droplets	Poor seedling vigour (plants showing poor growth and weak seedlings)	Nonglossy (dark green, dull, broad and drooping leaves)	Leaf sheath with green colour
6 (S)	-	25.1–30	-	-	-	-
7 (S)	-	30.1–35	-	-	-	-

^**a**^The 1–5 scale for scoring shoot fly damage in maize was calculated by converting percentages of the leaf injury into the rating score whereas the percentage of the dead hearts was classified into scale of 1–7. Leaf injury and deadheart counts were recorded thrice at an interval of 7 days i.e. at 7 days after emergence (DAE), 14 DAE and 21 DAE and were expressed in terms of percentage. The data on leaf glossiness, leaf sheath pigmentation, seedling vigor, and leaf surface wetness were recorded at 5 leaf stage of seedlings on the scale of 1–5

^b^The observations on leaf surface wetness were recorded between 7.00 to 7:30 A.M.

^c^Leaf glossiness was evaluated in the early morning hours when there was maximum reflection of light from the leaf surfaces

^d^The scale of 1–5 was categorized as: R-resistant, MR-moderately resistant, MS-moderately susceptible, S-susceptible

### 2.3 Molecular marker analysis

Genomic DNA was isolated from young seedlings of 107 F_2_ individuals and parents (CM143 and CM144) using the standard CTAB procedure [24]. *In vitro* amplification using polymerase chain reaction (PCR) was performed in a 96-well microplate in an Eppendorf^TM^ Master Cycler in 10 μl reaction volume as described by Kaur *et al* [25]. The PCR products were resolved in 3% 0.5X TBE agarose gel and the bands were visualized under the UVP gel documentation system. A total of 701 simple sequence repeats (SSR) markers curated from maize database (http://www.maizegdb.org), covering all regions of 10 linkage groups spanning all bins were selected for documentation of polymorphism between the parental lines. The F_2_ population was genotyped employing 199 polymorphic SSR markers and genetic map was constructed using 120 SSR markers.

### 2.4 Statistical analysis

Field data were subjected to analysis of variance as per standard procedure of randomized complete block design. Pearson correlation coefficients were calculated to establish association between different component traits. Linkage map was constructed with a threshold value of LOD score of 3.0 and recombination fraction of 0.3 using Mapdisto version 1.7.7 [26]. The QTLs were identified by composite interval mapping (CIM) using both forward and backward regression method with Windows QTL cartographer version 2.5 [27]. The threshold LOD was calculated using 1,000 permutations in each case with 5 per cent level of significance. The proportion of observed phenotypic variance explained by a QTL was estimated using coefficient of determination (*R*^2^) using maximum likelihood for CIM. Gene action for each QTL was calculated as per Stuber *et al* [28]. Briefly, values of 0 to 0.20 were taken for additive gene action, 0.21 to 0.80 as partial dominance, 0.81 to 1.20 as dominance and >1.20 as over dominance. The source of resistance allele was designated as per Jampatong *et al* [29]. Positive and negative additive values depicted that alleles were contributed by resistant parent CM143 and susceptible parent CM144, respectively.

### 2.5 *In silico* analysis

The potential candidate genes within the detected QTLs were fetched out from genome sequence of *Zea mays* (https://www.maizegdb.org/). Functional annotation of the downloaded genes was retrieved from maizegdb and the published literature. Blast2Go software was used for gene ontology analysis. The QTL-bearing sequences associated with shoot fly resistance revealed in the present study were compared to sorghum genome using NCBI blast to identify the syntenic regions between two genomes. Syntenic regions were plotted using CIRCOS program [30].

## 3. Results and discussion

### 3.1 Genetic basis of shoot fly resistance

#### 3.1.1 Inheritance of shoot fly resistance

**Fig 1:**
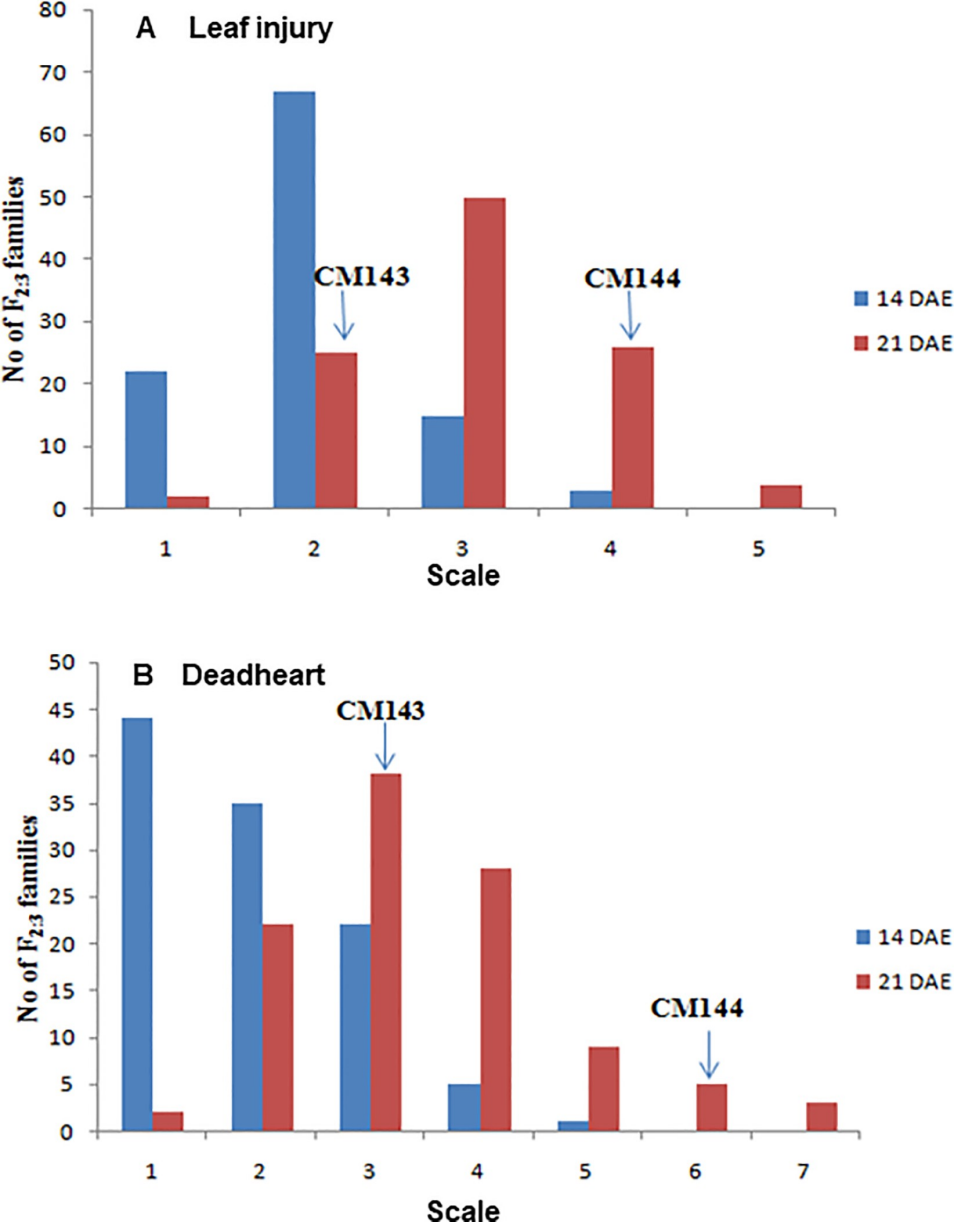
Distribution of leaf injury and deadheart at 14 and 21 days after emergence (DAE) for shoot fly resistance among F_2:3_ families derived from cross of CM143 × CM144. The 1–5 scale for shoot fly damage in F_2:3_ families was calculated by converting percentages of the leaf injury into the rating score (A), whereas the percentage of the dead hearts was classified into scale of 1–7 (B). Leaf injury scale- 1: 0–15%, 2: 15.1–25%, 3: 25.1–35%, 4: 35.1–45%, 5: 45.1–55%. Deadheart scale- 1: 0–5%, 2: 5.1–10%, 3: 10.1–15%, 4: 15.1–20%, 5: 20.1–25%, 6: 25.1–30%, 7: 30.1–35%. The position of the average scores of the parental types, CM143 and CM144, are indicated (Refer Table 2).

**Fig 2:**
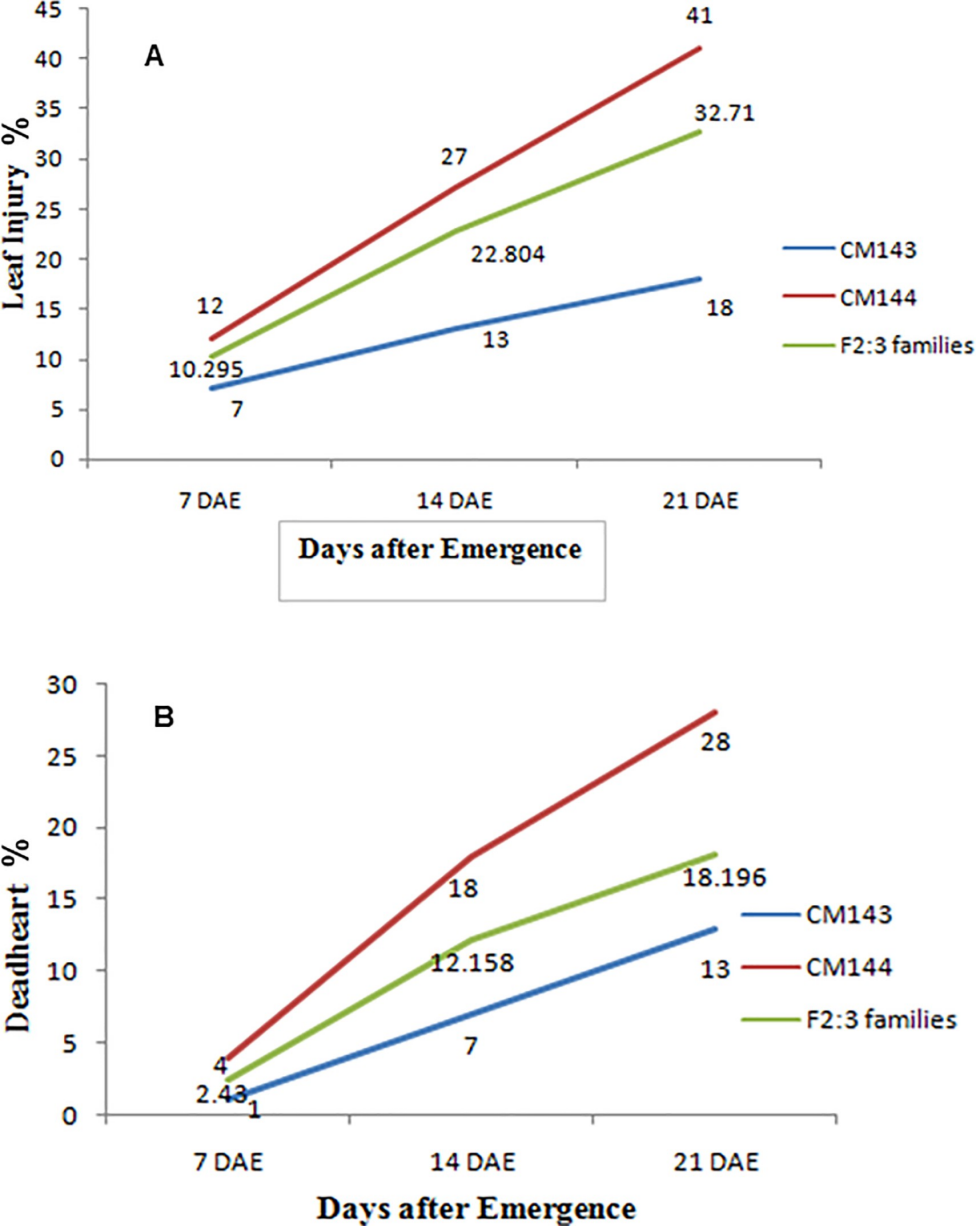
Leaf injury % (A) and deadheart % (B) progress curve of CM143, CM144 and F_2:3_ families derived from cross of CM143 × CM144 at 7, 14 and 21 days after emergence (DAE) after shoot fly infestation. The values represent the mean value of the parents and F_2:3_ families (Refer Table 2).

Plant morphology has a strong impact on shoot fly damage, especially seedling characteristics that physically reduce feeding, oviposition, and shelter. The phenotypic trait means of the parental lines and their F_2:3_ families for various component traits are presented in Tables 2 and 3. All the component traits showed significant differences between the parental lines and among the F_2:3_ families. Resistant parent CM143 recorded mean EC of 6.0, 13.0 and 20.0, whereas susceptible parent CM144 registered an average EC of 9.5, 21.0 and 38.5 at 5, 10 and 15 DAE, respectively indicating that a greater number of eggs was laid on susceptible parent CM144. The distribution of LI and DH data at 21 DAE revealed that the response of F_2:3_ families followed a normal curve with transgressive segregation in both directions (Fig 1). Moreover, different genotypes may vary in expression of injury depending upon their inherit ability to tolerate the damage. Similar observations were made for other traits. This indicated that variables for shoot fly resistance exhibited polygenic inheritance. The shoot fly severity progressed with time as indicated from LI and DH data (Fig 2). It could be inferred that the insect attack severity increases at 21 DAE and continues till the favourable conditions persists.

**Table 2:** Means and range of parents and F_2:3_ families derived from cross of CM143 × CM144 at different days after emergence (DAE) for egg count, leaf injury (%) and deadheart (%) after shoot fly infestation.

Parents/ Population	Oviposition (Egg count, EC)^a^					
	5 DAE^a^		10 DAE		15 DAE	
	Range	Mean	Range	Mean	Range	Mean
CM143	0–8	6.0±0.12	0–15	13.0±0.23	10–25	20.0±0.21
CM144	0–13	9.5±0.22	5–25	21.0±0.11	20–50	38.50±0.19
F_2:3_ families	0–11	7.64±0.13	10–25	17.59±0.08	17–43	28.36±0.16
			CD^d^	5.186	CV^e^	9.3
	**Leaf injury% (LI)**^b^					
	7 DAE		14 DAE		21 DAE	
CM143	0–10	7.0±0.114	0–15	13.0±0.167	15.0–25.0	18.0±0.104
CM144	0–15.0	12.0±0.126	25.0–35.0	27.0±0.118	35.0–45.0	41.0±0.234
F_2:3_ families	0–20.0	10.295±0.132	10–45.0	22.804±0.23	15.0–55.0	32.71±0.194
			CD	12.492	CV	19.24
	**Deadheart% (DH)**^c^					
	7 DAE		14 DAE		21 DAE	
CM143	0–5.0	1.0±0.216	5.0–10	7.0±0.109	10.0–15.0	13.0±0.134
CM144	0–10.0	4.0±0.109	15.0–20.0	18.0±0.094	25.0–30.0	28.0±0.122
F_2:3_ families	0–10.0	2.425±0.301	5.0–25.0	12.158±0.134	10.0–35.0	18.92±0.137
			CD	10.734	CV	31.97

^a^Egg count was recorded at an interval of 5 days i.e. at 5 days after emergence (DAE), 10 DAE and 15 DAE

^b&c^Leaf injury and deadheart were recorded at an interval of 7 days i.e. at 7 DAE, 14 DAE and 21 DAE and were expressed in terms of percentage

^d^CD: Critical difference

^e^CV: Coefficient of variation

**Table 3:** Mean values of parents and F_2:3_ families derived from the cross of CM143 × CM144 for various component traits after shoot fly infestation.

Traits^a^/ Lines	Seedling vigor (SV)	Leaf glossiness (LG)	Leaf sheath pigmentation (LSP)	Leaf surface wetness (LSW)	Leaf length (LL; cm)	Leaf width (LW; cm)	Leaf area (LA; cm^2^)	Stem girth (SG; cm)
CM143	1.5±0.137^d^	2.0±0.216	1.0±0.122	2.0±0.164	11.56±0.112	1.69±0.198	19.53±0.13	2.07±0.205
CM144	4.25±0.836	4.5±0.228	4.0±0.202	4.5±0.181	13.04±0.166	1.87±0.12	24.38±0.20	1.76±0.08
F_2:3_ families	2.18±0.195	2.71±0.058	1.66±0.311	3.28±0.115	12.001±0.175	1.711±0.123	20.53±0.124	1.98±0.177
CD^b^	1.333	1.028	1.001	1.094	2.098	0.379	8.157	4.76
CV^c^	30.78	19.15	30.22	16.82	7.54	10.56	15.64	11.54

^a^The data on SV, LG, LSP, LSW, LL, LW, LA and SG were recorded at 5^th^ leaf stage of seedlings. The data on SV, LG, LSP and LSW were recorded on the scale of 1–5. LG was evaluated in the early morning hours when there was maximum reflection of light from the leaf surfaces whereas the observations on LSW were recorded between 7.00 to 7:30 A.M

^b^CD: Critical difference

^c^CV: Coefficient of variation

^d^± Value is standard error of difference

High degree of positive association between EC and DH (r = 0.936); LI and EC (r = 0.891); LI and DH (r = 0.824); and between LG and LSW (r = 0.83) were observed (Table 4). The present results suggested that LI and DH are the major contributors to shoot fly damage because the eggs are laid by shoot fly on the leaves of emerging maize seedlings and subsequently growing tip is damaged leading to DH. The ability to recover from leaf injury is key for expression of resistance in a genotype. Both these traits are directly associated with the EC of shoot fly which is related or dependent on LG, and SV. It has been reported earlier that higher values of LL, LW, LA, and SG were associated with susceptibility to shoot fly in maize [2]. Similar results have been observed in the present study that the traits like LL, LW, LA, and SG were negatively associated with resistance (Table 4). Also, LL, LW, LA, and SG were non-significantly related with other component traits for shoot fly resistance. The leaf glossiness (light green and shiny leaves), leaf surface wetness (reduces the movement of freshly hatched larvae) and leaf sheath pigmentation (dark pink) at seedling stage has a strong influence on the oviposition of shoot fly and these traits were positively associated with oviposition and deadheart in the present work which are in agreement with Dhillon *et al* [22] but differ with the results of Satish *et al* [9] in sorghum. Also, association of seedling vigor with oviposition and deadheart was positive in present study but negatively correlated in sorghum as reported by Satish *et al* [9]. This suggests that the genetic relationship between these traits depends on the germplasm being evaluated and the differences may exist at genus level. Also, the inherent mechanisms of tolerance of plants drive the reaction either towards resistance or susceptibility.

**Table 4:** Correlation coefficients among various component traits for shoot fly resistance among F_2:3_ families derived from the cross of CM143 × CM144.

Traits	Leaf glossiness	Leaf surface wetness (LSW)	Leaf sheath Pigmentation	Leaf length (LL)	Leaf width (LW)	Leaf area (LA)	Stem girth (SG)	Leaf injury (LI^a^)	Deadheart (DH^b^)	Oviposition (EC15^c^)
	**(LG)**									
			**(LSP)**							
**Seedling vigor (SV)**	0.752	0.739	0.709	-0.103	-0.087	0.008	-0.008	0.669	0.669	0.668
	(< 0.0001)^d^	(< 0.0001)	(< 0.0001)	(0.289)	(0.373)	(0.932)	(0.935)	(< 0.0001)	(< 0.0001)	(< 0.0001)
**Leaf glossiness (LG)**		0.831	0.725	-0.068	-0.079	0.042	0.088	0.737	0.692	0.738
		(< 0.0001)	(< 0.0001)	(0.489)	(0.417)	(0.664)	(0.368)	(< 0.0001)	(< 0.0001)	(< 0.0001)
**Leaf surface wetness (LSW)**			0.694	-0.121	-0.166	-0.023	0.079	0.769	0.701	0.752
			(< 0.0001)	(0.215)	(0.088)	(0.810)	(0.419)	(< 0.0001)	(< 0.0001)	(< 0.0001)
**Leaf sheath pigmentation (LSP)**				-0.165	-0.183	-0.099	0.040	0.702	0.658	0.702
				(0.090)	(0.060)	(0.309)	(0.684)	(< 0.0001)	(< 0.0001)	(< 0.0001)
**Leaf length (LL)**					0.736	0.854	0.342	-0.027	0.022	-0.019
					(< 0.0001)	(< 0.0001)	(0.001)	(0.783)	(0.819)	(0.849)
**Leaf width (LW)**						0.855	0.318	-0.035	-0.010	-0.036
						(< 0.0001)	(0.001)	(0.720)	(0.918)	(0.712)
**Leaf area (LA)**							0.371	0.113	0.131	0.100
							(< 0.0001)	(0.248)	(0.180)	(0.307)
**Stem girth (SG)**								0.217	0.148	0.204
								(0.024)	(0.129)	(0.035)
**Leaf injury (LI)**									0.824	0.891
									(< 0.0001)	(< 0.0001)
**Deadheart (DH)**										0.936
										(< 0.0001)

^a^EC: Egg count data at 15 days after emergence (DAE) was used for analysis

^b^LI: Leaf injury data at 21 DAE was permutated for analysis

^c^DH: Deadheart data at 21 DAE was analyzed

^d^The value in parenthesis indicates the significance probability associated with the statistic

#### 3.1.2 QTL mapping

**Fig 3:**
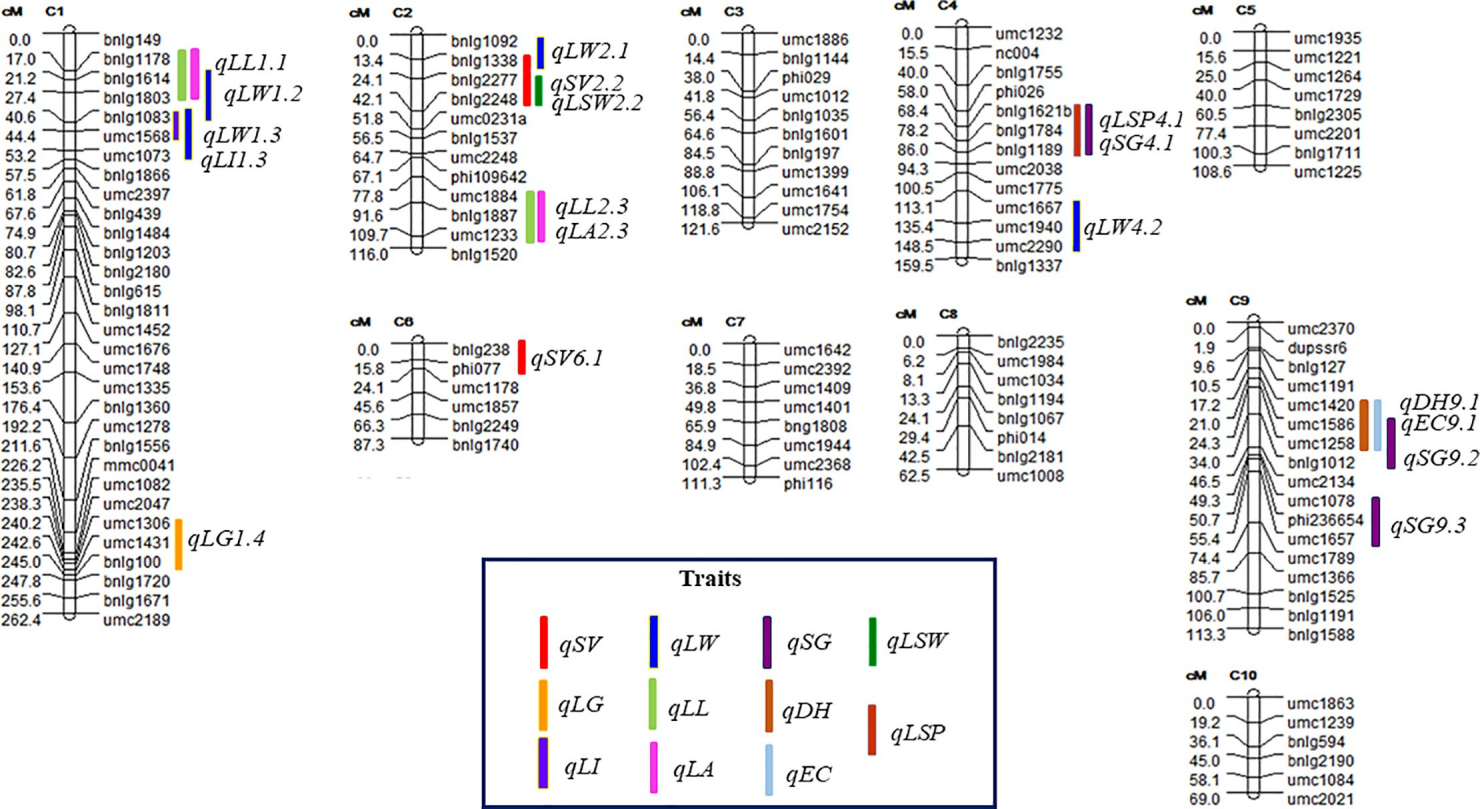
Genetic linkage map of maize representing 19 quantitative trait loci (QTL) for shoot fly resistance identified in F_2:3_ families derived from cross of CM143 × CM144. Putative QTL are designated by the corresponding chromosome in which they are found. The map distance is given on the left in centimorgans (cM) from the top of each chromosome and marker name is represented on the right side of the chromosome. Different color bars are used to indicate QTL for each trait. LL: Leaf length, LW: Leaf width, LA: Leaf area, LI: Leaf injury, LSW: Leaf surface wetness, LSP: Leaf sheath pigmentation, LG: Leaf glossiness, SV: Seedling vigor, SG: Stem girth, EC: Oviposition, DH: Deadheart.

A total of 701 maize SSR markers were surveyed for parental polymorphism and 228 (32.52%) markers were found to be polymorphic. A set of 199 polymorphic SSR markers were genotyped on F_2_ population. Significant deviation from expected Mendelian segregation was observed for 74 SSR markers (37.18%); 21 and 18 SSR markers were skewed towards CM143 and CM 144, respectively. Whereas, 30 markers had banding pattern more towards heterozygosity. Segregation distortion might be due to gamete abortion or the selective fertilization of particular gametic genotypes, presence of segregation-distortion loci (SDL) in the vicinity of markers, epistasis, enhanced recombination [31]. In the present investigation five of the SSR markers showed banding pattern resembling either of the parents in the whole population. This might be due to the presence of ‘cold spot regions’ showing little or no recombination [32] or due to a gamete elimination system [33]. All these markers were excluded from data analysis. Five of the SSR markers did not show linkage with their respective linkage group during the development of genetic linkage map. The relative position and genetic distances of 120 SSR markers along the respective chromosome are presented in Fig 3. The genetic map had a total length of 1211.5 cM with an average distance of 10.1 cM between markers. The order of the markers on the linkage map agreed with the corresponding positions on the IBM 2008 map (https://www.maizegdb.org/) except four markers (*umc1452*, *bnlg1866*, *bnlg615*, and *umc2248*) whose chromosomal location agreed but not specified according to the bin position.

Numerous studies have been conducted to map QTLs for resistance to European corn borer, Mediterranean corn borer, Asian corn borer, sugarcane borer, maize weevil, Southwestern corn borer, and fall army worm [34]. However, no information is available about the number, mode of action, and location of QTLs governing shoot fly resistance in maize. A total of 19 putative QTLs associated with various component traits governing shoot fly resistance were detected on chromosomes 1, 2, 4, 6, and 9 (Table 5). It has been observed that resistant as well as susceptible parent contributed alleles for shoot fly resistance as none of the parents was completely resistant or susceptible to shoot fly. Nine (*qLW4*.*2*, *qLL2*.*3*, *qLA2*.*3*, *qLSW2*.*2*, *qSV2*.*2*, *qEC9*.*1*, *qDH9*.*1*, *qSG9*.*2*, and *qSG9*.*3*) of the 19 QTLs alleles conferring resistance to the shoot fly in the present investigation were contributed by the susceptible parent, CM144, whereas positive alleles of other 10 QTLs were contributed by the resistant parent, CM143. Other authors have also reported QTL alleles for insect resistance that came from a highly susceptible parent in maize [11, 16], rice [35] and cotton [36]. It indicates that susceptible parent also harbor some favorable alleles, which can be employed in gene pyramiding.

**Table 5:** Marker intervals showing putative QTL for shoot fly resistance component traits in F_2_ population from cross of CM143 × CM144.

Trait	QTL^a^	Marker interval	Bin^b^	LOD score^c^	Phenotypic variance^d^ (%)	Additive effect^e^	Dominance effect^f^	Gene Action^g^
Leaf width	*qLW1*.*2*	*bnlg1614-bnlg1083*	1.02	3.68	9.32	0.0526	0.0314	PD
	*qLW1*.*3*	*bnlg1083-umc1073*	1.02–1.03	4.27	9.94	0.0595	0.0194	PD
	*qLW2*.*1*	*bnlg1092-bnlg1338*	2.01	2.65	8.16	0.05	0.0031	A
	*qLW4*.*2*	*umc1667-umc2290*	4.08	3.18	11.26	-0.0457	0.0312	PD
Leaf length	*qLL1*.*1*	*bnlg1178-bnlg1803*	1.02	4.67	4.25	0.1162	0.2853	OD
	*qLL2*.*3*	*bnlg1884-umc1233*	2.05	2.94	10.85	-0.1465	0.0236	A
Leaf area	*qLA1*.*1*	*bnlg1178-bnlg1803*	1.02	6.28	8.48	0.3731	0.6358	OD
	*qLA2*.*3*	*umc1884-umc1233*	2.05	2.55	9.82	-0.3392	0.1158	PD
Leaf injury	*qLI1*.*3*	*bnlg1083-umc1568*	1.02	3.14	11.96	0.1143	-	A
Leaf surface wetness	*qLSW2*.*2*	*bnlg2277-bnlg2248*	2.02–2.03	3.22	7.30	-0.018	-	A
Leaf glossiness	*qLG1*.*4*	*umc1306-bnlg100*	1.09	2.62	12.98	0.0501	-0.0614	D
Leaf sheath pigmentation	*qLSP4*.*1*	*bnlg1621b-bnlg1189*	4.07	3.08	7.58	0.1207	0.0439	OD
Seedling vigour	*qSV2*.*2*	*bnlg1338-bnlg2248*	2.01–2.03	2.73	9.8	-0.0432	0.1402	OD
	*qSV6*.*1*	*bnlg238-phi077*	6.00–6.01	2.80	9.7	0.0340	-0.1229	OD
Oviposition	*qEC9*.*1*	*umc1420-umc1258*	9.03	4.09	18.49	-2.881	0.8197	OD
Dead heart	*qDH9*.*1*	*umc1420-umc1258*	9.03	3.49	15.03	-0.0393	0.0040	A
Stem girth	*qSG9*.*2*	*umc1586-bnlg1012*	9.05	2.65	7.26	-0.1722	-0.2282	OD
	*qSG9*.*3*	*umc1078-umc1657*	9.06	4.27	10.5	-0.1118	-0.5880	OD
	*qSG4*.*1*	*bnlg1621b-bnlg1189*	4.07	3.47	9.8	0.0670	-0.3669	OD

^a^Putative QTL are designated by the corresponding chromosome in which they are found

LL: Leaf length, LW: Leaf width, LA: Leaf area, LI: Leaf injury, LSW: Leaf surface wetness, LSP: Leaf sheath pigmentation, LG: Leaf glossiness, SV: Seedling vigor, SG: Stem girth, EC: Oviposition, DH: Deadheart

^b^Chromosome bin location of QTL peak of the maize genome. Bins divide the genetic map into 100 approximately equal segments of approximately 20 centiMorgans between two fixed Core Marker. The segments are designated with the chromosome number followed by a two-digit decimal (e.g., 1.00, 1.01, 1.02, etc)

^c^The maximum LOD score associated with each QTL

^d^*R*^2^ estimates the proportion of phenotypic variance (%) explained by individual QTL

^e^The additive genetic effect of the putative QTL. A positive number indicates that the alleles for resistance are derived from CM143 and a negative number means that the alleles for resistance are derived from CM144

^f^The dominant genetic effect of the putative QTL

^g^Gene action displayed by a QTL: A (additive) = > 0.20, PD (partial dominance) = 0.21 to 0.80, D (dominance) = 0.81 to 1.20, OD (over dominance) = > 1.20

Four QTLs for leaf width (*qLW1*.*2*, *qLW1*.*3*, *qLW2*.*1* and *qLW4*.*2*) depicting partial-dominance and additive gene action were identified on short arm of chromosome 1 (bins 1.02–1.03), chromosome 2 (bin 2.01) and long arm of chromosome 4 (bin 4.08) explaining 38.68% of total phenotypic variance among F_2:3_ families. Most of the alleles associated with leaf width were contributed by the resistant parent, CM143. The QTLs for leaf length and leaf area present on short arm of chromosome 1 (*qLL1*.*1* & *qLA1*.*1*) revealed over dominance gene action whereas, the *qLL2*.*3* and *qLA2*.*3* present on long arm of chromosome 2 displayed partial dominance and additive gene action, respectively.

**Fig 4:**
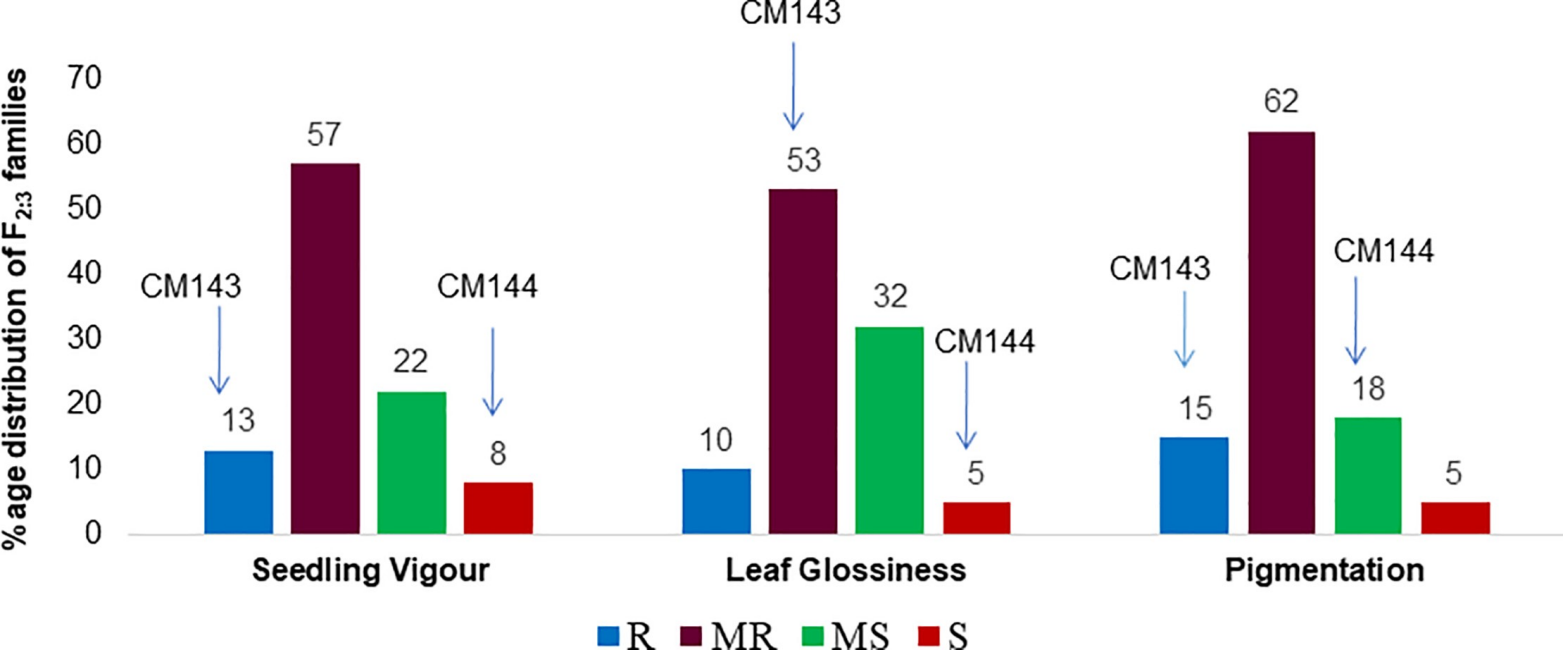
Per cent distribution of F_2:3_ families derived from cross of CM143 × CM144 into different categories with respect to seedling vigor, leaf glossiness and leaf sheath pigmentation. The data on these traits were recorded at 5 leaf stage of seedlings on the scale of 1–5. The scale of 1–5 was categorized as: R-resistant = 1, MR-moderately resistant = 2, 3, MS-moderately susceptible = 4, S-susceptible = 5. The number on the bar indicates the percentage of F_2:3_ families into that category. The position of the average scores of the parental types, CM143 and CM144, are indicated.

The QTL for LI and LG were detected in different genomic regions of chromosome 1 (short arm, bin 1.02 and long arm, bin 1.09) accounting for 11.96 and 12.98% phenotypic variation, respectively (Fig 3 and Table 5). Gene action effects were additive and dominant for LI and LG, respectively. Two QTL, *qSV2*.*2* and *qSV6*.*1*, for seedling vigor exhibiting overdominance were detected on short arm of both chromosomes 2 and 6 with favourable alleles contributed by CM144 and CM143, respectively. Overdominance gene action was exhibited by all the three QTL for SG present on long arm of chromosome 4 (*qSG4*.*1*) and 9 (*qSG9*.*2*, *qSG9*.*3*) accounting for 27.56% phenotypic variance together (Table 5). Alleles associated with reduced SG were contributed by susceptible parent, CM144. Most of the QTLs for insect resistance in maize showed additive gene action [16, 21]. However, present study depicted that maize shoot fly resistance was governed by overdominance (nine QTLs), dominance (one QTL), partial dominance (four QTLs) and additive (five QTLs) type of gene action. It indicates that genetics of shoot fly resistance is complex and involves different types of gene action for different component traits. Similar results were reported by Garcia-Lara *et al* [11] that genetic effects were mainly dominant and additive for maize weevil resistance. Our results also support that leaf glossiness was positively correlated with the level of resistance as 53 per cent of F_2:3_ families were moderately resistant and the allelic contribution for leaf glossiness was inherited from the female parent (CM143) in dominant manner. Similarly, leaf sheath pigmentation and seedling vigor traits showed overdominance expression as specified from maximum number of F_2:3_ families were resistant and moderately resistant (Fig 4).

The detected QTLs for shoot fly component traits co-localized in seven genomic regions located on chromosomes 1, 2, 4 and 9 (Fig 3). Two QTLs (present on chromosome bins 1.02 and 2.05) each for leaf length (*qLL1*.*1*, *qLL2*.*3*) and leaf area (*qLA1*.*1*, *qLA2*.*3*) were co-localized (Fig 3) as both traits are positively correlated. The QTL *qLW1*.*3* present in the genomic region *bnlg1083-umc1073* overlapped with *qLI1*.*3*. Likewise, leaf sheath pigmentation QTL, *qLSP4*.*1*, was co-localized to stem girth QTL, *qSG4*.*1*, on chromosome 4 whereas seedling vigor QTL, *qSV2*.*2* (R^2^ = 9.8%) was overlapped with leaf sheath pigmentation QTL, *qLSW2*.*2* (R^2^ = 7.3%).

The QTL for deadheart (*qDH9*.*1*) spanning the marker interval of *umc1420* and *umc1258* (9.03) explained 15.03% of phenotypic variance. This putative QTL was co-localized with oviposition QTL (*qEC9*.*1*) with 18.89% of phenotypic variance (S3 Fig). This QTL was residing near the centromeric region of chromosome 9. Our results are in concordance to Satish *et al* [9] and Apotikar *et al* [37] regarding co-localization of EC and DH QTLs in sorghum chromosome 10. So, this region could be further dissected to identify the candidate genes for shoot fly resistance in maize. Co-localization of QTLs for different traits might be result of tight linkage of several genes (cluster of genes are present in the form of multigene family controlling different traits) or pleiotropic effect of a locus [9].

The previous studies concluded that disease and insect resistance genes in maize appear in “clusters” [38]. The detected bins in the experiment (1.02–03, 1.09, 2.05, 6.00–6.01, 9.03 and 9.05–06) have been reported in different studies for insect resistance especially to European corn borer, fall army worm and maize weevil as presented in S1 Table. Based on meta QTL analysis, Badji *et al* [34] highlighted the presence of combined insect resistance genomic regions in maize and thus, could form the basis for multiple pest’s resistance breeding. This clearly supports the hypothesis that there are certainly some common genomic regions that confer resistance against insect pests along with specified genomic regions unique to specificity for each of the pests. Hence, these common genomic regions are key for further studies to dissect the resistance genes and might enable for deciphering the common metabolic pathway conferring insect pest resistance.

### 3.2 Resistance mechanism

Plant resistance to insect herbivory can be classified as antixenosis (non-preference of host plants), antibiosis, and tolerance [1]. Non-preference by insects is often projected as a property of the plant to render it unattractive for oviposition, feeding, and shelter. The antixenosis for oviposition by shoot fly was not observed in maize genotypes and the tolerance was mainly dependent on the ability of plant to recover from injury [2, 7]. Similar observations were recorded in the present investigation. However, the differences in oviposition exists that may be due to aggregation distribution of eggs at high density or may be due to antixenosis. Scanty information is available on the mechanism of antibiosis for resistance to shoot fly.

The QTLs for leaf dimensions and leaf injury were detected in bin 1.02 in the present study. In an earlier investigation by Barriere *et al* [39], cell wall-bound phenolic compounds (p-coumaric acid, esterified ferulic acid etc.) were found to be associated in bin 1.01/1.02. Similarly, the identified QTL in bin 1.09, 2.01, 2.05, 4.08, 6.01, 9.03, 9.05–9.06 also included QTL for different metabolites (maysin, chlorogenic acid, Feuric acid, p-coumaric acid, DIMBOA and diferuloyl putrescine) as detected in other experiments [40–42]. It is well known that the cell wall constituents act as a barrier to feeding insects and its proximal co-localization with shoot fly component trait QTLs validates the importance of cell wall structure and composition in resistance. It has been reported earlier that QTLs for leaf feeding & stem feeding co-localized with cell wall constituents in 29 maize bins [1]. It could be concluded that antibiosis increases the mortality and hampers the growth and feeding of larvae on the host plant. As there is close liaison among QTLs linked with shoot fly resistance and cell wall components, it will be a rewarding exercise to map QTLs for these metabolites using the same mapping population.

**Fig 5:**
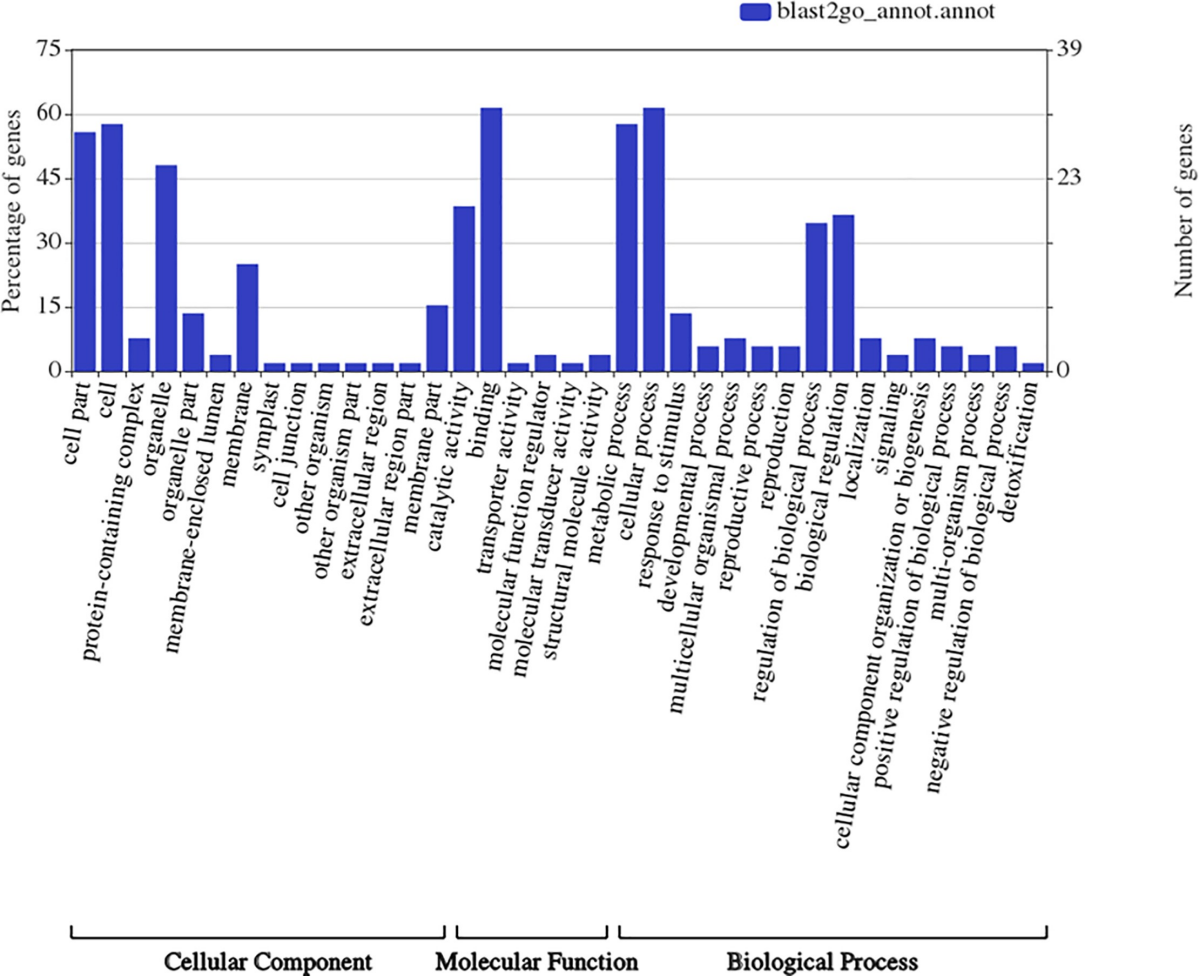
Gene ontology classification of the genes identified within the detected QTL regions for shoot fly resistance.

In the present study, the number of genes detected in the QTL regions varied from 91 to 295. The putative 58 candidate genes were short-listed from a previous study by Satish *et al* [9] and on the basis of their role in plant stress response. These genes were categorized into receptor kinases (leucine-rich repeat receptor kinase, serine/threonine protein kinases and sucrose non-fermenting related protein kinases 1), signalling molecules or secondary messengers, metabolic enzymes, transcription factors and some proteins acting as chaperons (S2 Table). Most of the candidate genes are involved in jasmonic signalling pathway, lignin biosynthesis, meristem growth, and biotic and abiotic stress tolerance. These genes were classified into three categories namely, cellular function, molecular function and biological process according to gene ontology (GO) using Blast2Go (Fig 5). GO analysis of cellular components showed that genes were associated with all parts of the cell. In the molecular function category, catalytic activity and various binding activities genes were enriched. In biological process, most of the genes were associated with cellular process, metabolic process, biological regulation and regulation of biological process. It can be foreseen that crosstalk might be existing among different pathways involved in shoot fly resistance.

The insect oral components act as elicitors recognized by the plant to regulate the wounding response [43]. The endogenous resistance mechanism to shoot fly wounding is induced by the synthesis of proteinase inhibitors as evident from the QTLs spanning LG and SV traits harbour candidate genes for cysteine protease and subtilisin-chymotrypsin inhibitor on chromosome 2 and 6, respectively (S2 Table). The accumulation of the 33-kDa cysteine protease in the maize midwhorl was associated with a significant reduction in larval growth due to diminished nutrient utilization [44]. Similarly, subtilisin/chymotrypsin inhibitor gene (*Zm00001d035683*) detected on chromosome 6 also plays a role of natural defense against attacks of pests and pathogens [45]. This might be responsible for high SV associated with shoot fly resistance. The induced direct defenses also involve the production of toxic or repellent secondary metabolites and volatiles that attract natural enemies of the herbivore to the plant as indirect defense. The presence of putative genes for cytochrome P450s superfamily in the present study indicates that maize plants are involved in the production of allelochemicals, as P450s mediate the synthesis of allelochemicals [46]. The nature and type of allelochemical for the shoot fly resistance is yet to be elucidated.

The identified genes for GH10 family of glycoside hydrolases and extracellular sulfatases in the QTL interval of DH are present in extracellular space and have been implicated in the defense response by mobilization of energy reserves through degradation of starch and proteoglycan metabolism, respectively. These enzymes regulate signalling pathways by regulating the binding of signal ligands and receptor kinase [47, 48]. The genes for membrane bound receptor protein kinase have been identified within the QTL region of DH and EC, SG, and SV. All these have been implicated in initiating various signalling pathways, including meristem function, brassinosteroid perception, overall plant morphology, and plant defense [49]. These are further under regulation of phosphatases (PP2Cs) and ubiquitin/proteasome system. The genes for protein deubiquitination, ubiquitylation, protein-protein interaction, and proteasomal degradation (PP2C, Ubx2, ubiquitin C-terminal hydrolase, Speckle-Type Poz Protein (SPOP), and Ring finger zinc protein) have been localized within the QTL region of LG, SV, SG, and DH (S2 Table).

It has been reported that there is co-activation of numerous intracellular signalling cascades (Table 5). Jasmonic acid (JA) levels increase after wounding or attack by herbivores [50]. Fatty acid desaturase has been shown to trigger the JA defense signalling pathway which in turn regulates the induction of cysteine protease [51]. The gene for fatty acid desaturase identified in the present study is likely to be involved in imparting tolerance to shoot fly as gene for cysteine protease was also detected. Several studies have also shown that Ca^2+^ is an important messenger in many biotic and abiotic signals. Most of the Ca^2+^ sensors are proteins with atleast one highly conserved Ca^2+^ binding helix-turn-helix structures (EF-hands) and members of the MYB class of TFs [52]. The candidate genes for EF-hand MYB related transcription factors identified in the QTL interval of SV and LG traits provide a clue that Ca^2+^ signaling may play a role in shoot fly tolerance. Also, the gene for plasma membrane localized cyclic nucleotide gated channels (CNGCs) on chromosome 6 (*qSV6*.*1*; *bnlg238-phi077*) identified in the present work, has been reported to be involved in uptake of Ca^2+^ and other cations [53]. Likewise, a gene for fatty acid binding proteins present in QTL interval of *qSV6*.*1* have been identified in response to wound, it could be inferred that oral secretions of shoot fly might contain fatty acid conjugates which is sensed by the plants. It indicates that fatty acid binding proteins may be involved not only in intracellular signalling but also play a role in long-distance lipid signalling and may function as ‘chaperones’ which move lipids into and/or throughout the phloem [54].

In the present investigation, the genes identified for proline oxidase, nudix hydrolase, peroxidise, γ-glutamylcysteine, glutathione S-transferases and TETRATRICOPEPTIDE THIOREDOXIN-LIKE (TTL) have been reported to be involved in response to oxidative stress, redox balance regulation, and to modulate the levels of their substrates to maintain physiological homeostasis [55–58]. The identified genes for some of the transcription factors, including members of the C2H2 zinc finger protein (ZFP), HDZIP, NAC, MYB, Knotted1-like and bHLH families participate in plant stress response [59]. Most of the members of the Knox (Knotted1-like) class are associated with the maintenance and growth of the shoot meristems [60]. Both HD-ZIP class I (HAT7) and HD-ZIP class II (ATHB4, HAT14 & HAT22) respond to illumination sensing and phytochome-mediated shoot morphogenesis, respectively. Some of the HDZip I and II genes are involved in mediating the effects of external conditions to regulate plant growth and development [61].

Glossy phenotypes have less deposition of epicuticular wax and usually impart tolerance to insect damage. The QTL spanning LG in chromosome bin 1.09 contain some putative genes belonging to MYB transcription factors. Some MYB family transcription factors regulate the genes involved in wax biosynthesis and transport of cuticular components [62]. The candidate gene for MYB transcription factor identified for LG in the present study might be acting as negative regulator for wax biosynthesis. The previous studies demonstrated that wax biosynthesis genes were down regulated in the glossy mutant of Brassica [63] and by AP2/ERF type transcription factor in Arabidopsis [64]. The *gl15* (*glossy 15*) gene present in bin 9.03 was also reported as a candidate gene for fall army worm, south western corn borer and European corn borer resistance [19]. It alters the standard shift from vegetative to adult plant growth stage. This could be also one of the probable candidate genes associated with maize shoot fly resistance. From the present investigation, it could be inferred that tolerance in conjunction with antibiosis mechanism exists for shoot fly resistance in maize. Further studies are required to explore the metabolic signalling pathways for deciphering shoot fly resistance mechanism.

### 3.3 Syntenic relationships

**Fig 6:**
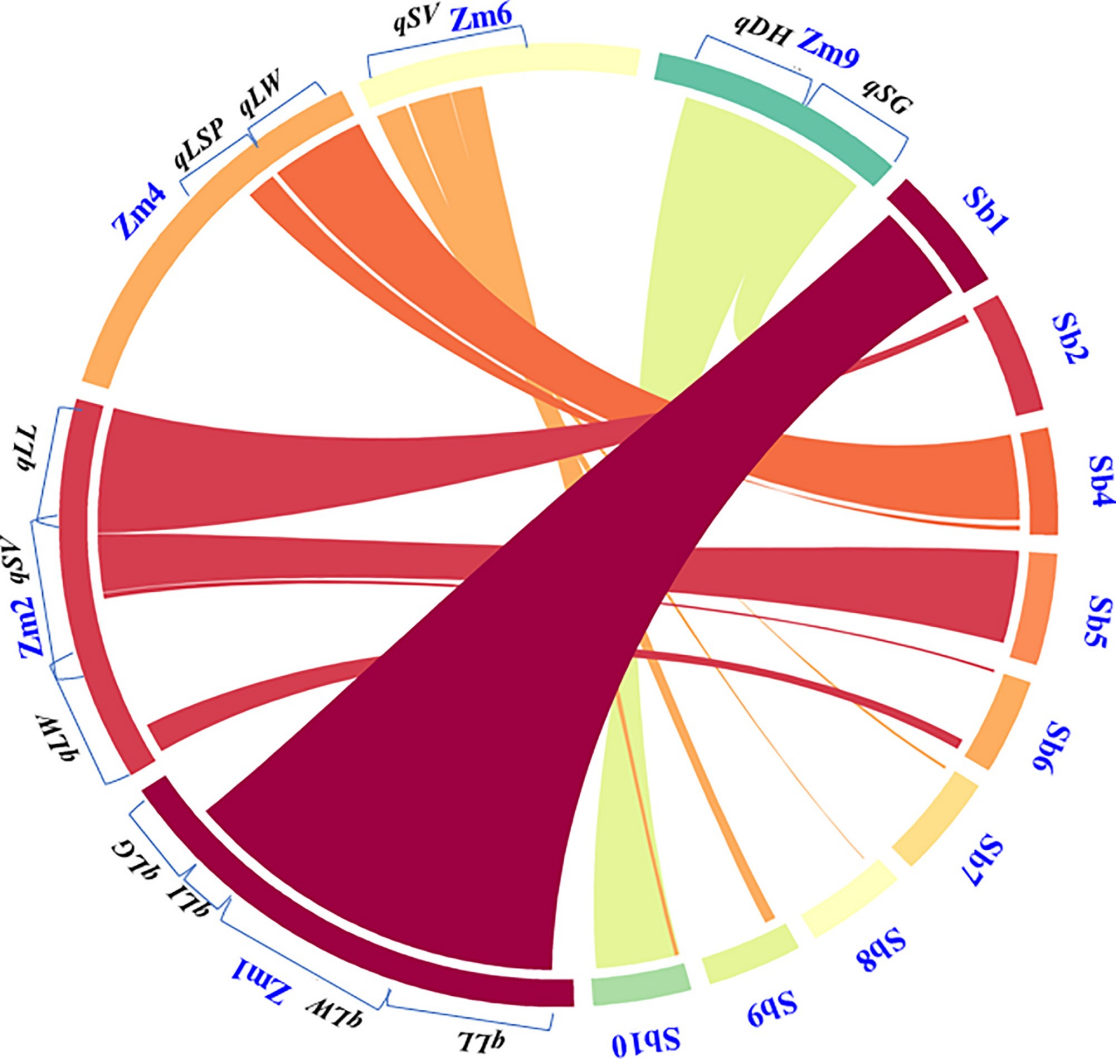
Syntenic relationship of maize genomic regions associated for shoot fly resistance component traits with sorghum genome. Zm1refers to *Zea mays* chromosome 1 and so on. Sb1 refers to *Sorgum bicolor* chromosome 1 and so on. LL: Leaf length, LW: Leaf width, LI: Leaf injury, LSP: Leaf sheath pigmentation, LG: Leaf glossiness, SV: Seedling vigor, SG: Stem girth, DH: Deadheart.

We investigated the syntenic relationships of the QTLs identified in the present study with those of reported in sorghum associated with shoot fly resistance [9]. Two major QTLs associated with DH between markers *Xnhsbm1044-Xnhsbm1013* and *Xnhsbm1033-Xcup16* on chromosome 10 explaining 15.0 and 11.4 per cent phenotypic variance, respectively in sorghum were found to be syntenic to bins 9.02–9.03 of maize chromosome 9 on which QTL for DH and EC were localized in the present study (Fig 6). The SV QTL located on maize chromosome 2 was found to be syntenic to SV QTL on chromosome 6 in sorghum. Similarly, SV QTL present on chromosome 6 of maize was coinciding to small genomic regions of sorghum chromosome 7, 8, 9 and 10. The QTLs (*qLL1*.*1*, *qLW1*.*2*, *qLI1*.*3*) identified on maize chromosome 1 and QTLs (*qSG9*.*2*, *qSG9*.*3*) located on chromosome 9 were syntenic to sorghum chromosome 1 where QTL for trichome density on lower leaf surface was detected by Satish *et al* [9]. Similarly, chromosome 4 (4.07–4.09) of maize harbouring QTLs for LW, SG, and LSP corresponded to sorghum chromosome 4 accounting for trichome density on lower leaf surface QTL. The foregoing results suggests that same gene blocks may be responsible for shoot fly resistance in both the cereal crops, sorghum and maize.

## Conclusion

Till date, all published QTL mapping studies on maize insect resistance has involved stem borers and storage pests. To the best of our knowledge, there is no report on the identification of QTLs for shoot fly tolerance in maize. We identified 19 QTLs on chromosomes 1, 2, 4, 6, and 9 in bins 1.02–1.03, 1.09, 2.01, 2.05, 4.07, 4.08, 6.00–01, 9.03 and 9.05–9.06 conferring tolerance to shoot fly. Many of the QTLs for component traits were found to be either overlapped or co-localized suggesting tight linkage of several genes. Also, the identification of co-localized QTL could be utilized for improvement of more than one trait at a time using the same linked markers. Some of the identified maize QTLs were observed to be syntenic with those of sorghum, thus confirming their association with shoot fly resistance. The transgressive segregants from the present material have been advanced and are being used in maize breeding programs. The identified regions of the QTLs need to be saturated with an additional set of markers, which will facilitate fine mapping of the QTLs. The present study has provided an insight in understanding the genetic basis of shoot fly resistance in maize.

## Supporting information

S1 Fig **A**: CM143 (moderately resistant) and **B**: CM144 (susceptible), infested with shoot fly after the seedling emergence using fish meal technique. **C**: Field view of F_2:3_ families derived from the cross of CM143 × CM144 (C).
(TIF)

S2 Fig Evaluation of different component traits on parents (CM143, CM144) and F_2:3_ families derived from the cross of CM143 × CM144 to shoot fly infestation on scale of 1 to 5. The data was recorded at different time intervals for different traits. **A**: Egg count on seedlings, **B**: Symptoms of leaf injury, **C1**: Highly vigorous seeding, **C5**: Poor seedling vigor, **D1**: Highly glossy, **D5**: Non-glossy, **E1**: Leaf sheath with dark pink pigment, **E5**: Leaf sheath with green color, **F**: Deadheart formation (refer Table 1).
(TIF)

S3 Fig QTL likelihood plot showing QTL for oviposition at 15 DAE and dead heart per cent at 21DAE peaks on chromosome 9 from analysis of data based on composite-interval mapping from F_2:3_ families derived from the cross of CM143 × CM144. The vertical axes in graph indicate LOD scores, and the horizontal line indicate the empirically derived LOD threshold for calling a QTL position. Small triangles on the x-axes denote the position of mapped SSR markers in the population and number represent the genetic distance in cM. One triangle may represent one or more markers in the case of very closely linked markers.
(TIF)

S1 Table Summary of location of insect pest resistance QTLs in maize. (DOCX)

S2 Table List of the short-listed putative candidate genes present in detected QTL intervals for shoot fly resistance in maize. (DOCX)
