# Supplementary figures and images for: Identification of genomic regions associated with shoot fly resistance in maize and their syntenic relationships in the sorghum genome
Source: PLoS One. 2020 Jun 9;15(6):e0234335. doi: 10.1371/journal.pone.0234335 (PMC7282634; doi:10.1371/journal.pone.0234335)

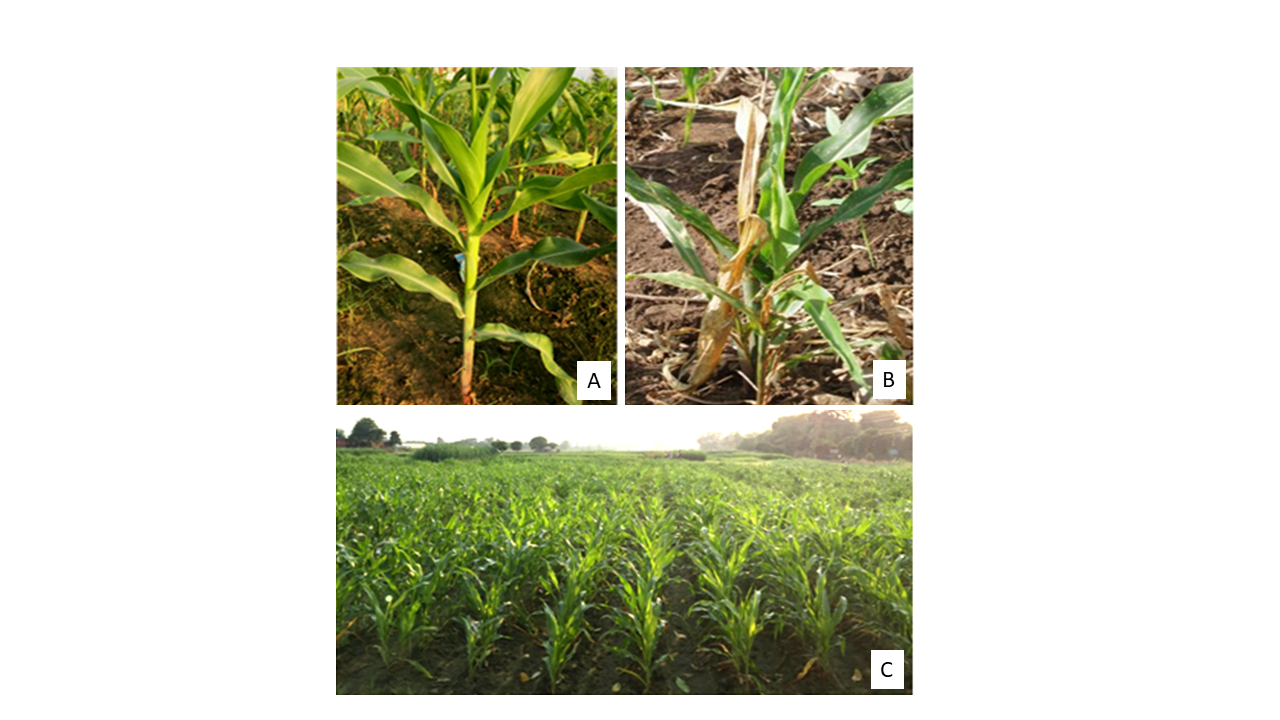

Supplement: S1 Fig — A: CM143 (moderately resistant) and B: CM144 (susceptible), infested with shoot fly after the seedling emergence using fish meal technique. C: Field view of F2:3 families derived from the cross of CM143 × CM144 (C). (TIF) [file pone.0234335.s001.tif]

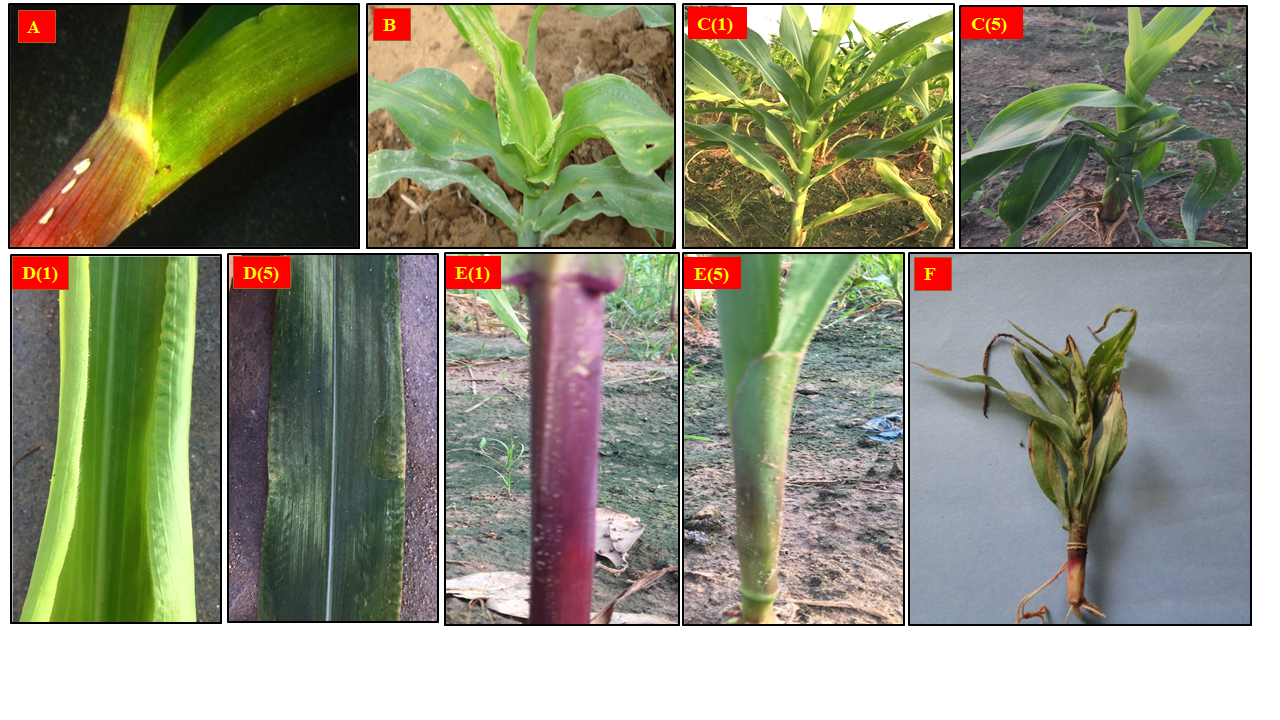

Supplement: S2 Fig — The data was recorded at different time intervals for different traits. A: Egg count on seedlings, B: Symptoms of leaf injury, C1: Highly vigorous seeding, C5: Poor seedling vigor, D1: Highly glossy, D5: Non-glossy, E1: Leaf sheath with dark pink pigment, E5: Leaf sheath with green color, F: Deadheart formation (refer Table 1). (TIF) [file pone.0234335.s002.tif]

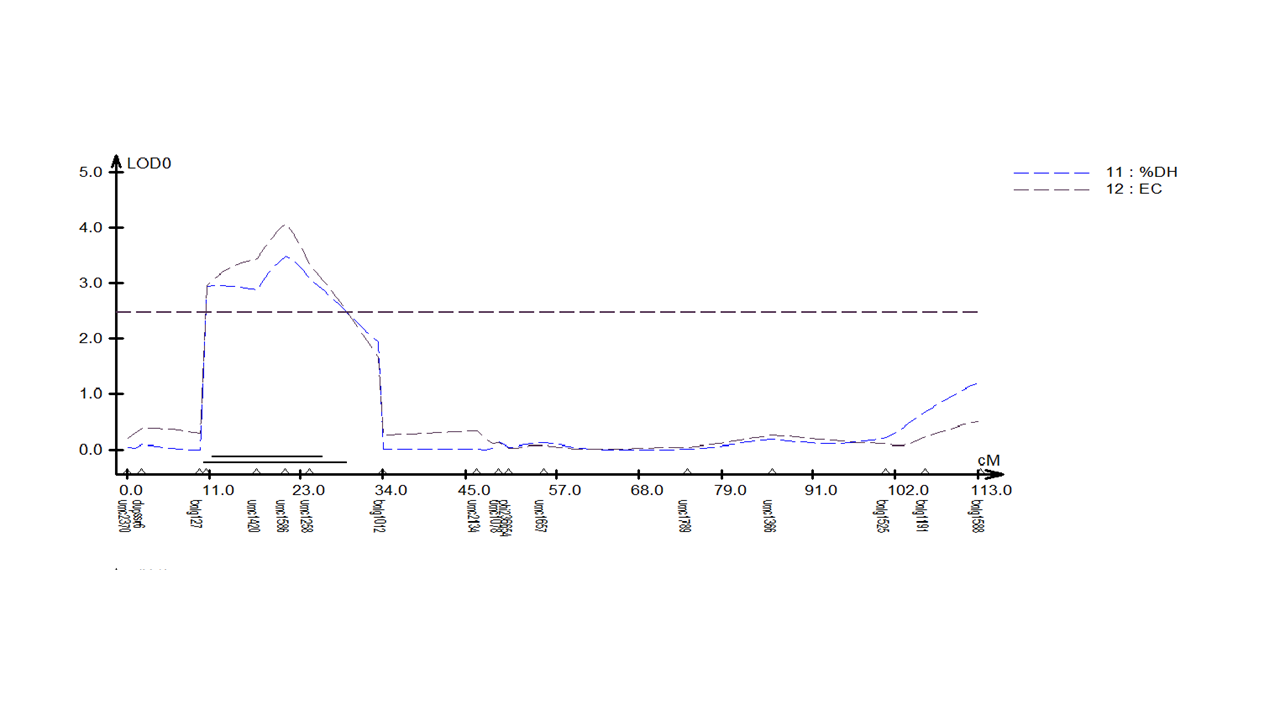

Supplement: S3 Fig — The vertical axes in graph indicate LOD scores, and the horizontal line indicate the empirically derived LOD threshold for calling a QTL position. Small triangles on the x-axes denote the position of mapped SSR markers in the population and number represent the genetic distance in cM. One triangle may represent one or more markers in the case of very closely linked markers. (TIF) [file pone.0234335.s003.tif]
