# Supplementary material for: Identification of genomic regions associated with shoot fly resistance in maize and their syntenic relationships in the sorghum genome
Source: PLoS One. 2020 Jun 9;15(6):e0234335. doi: 10.1371/journal.pone.0234335 (PMC7282634; doi:10.1371/journal.pone.0234335)
Supplement: S1 Table — (DOCX) [file pone.0234335.s004.docx]

**S1 Table.** **Summary of location of insect pest resistance QTLs in maize**

| **S. No** | **Bins Reporting QTLs^a^** | **Trait^b^** | **Populations^c^** | **References** |
| --- | --- | --- | --- | --- |
| 1 | 1.07-1.08, 1.10, 2.09, 5.00-5.01, 5.07-5.08, 6.01-6.02, 6.05-6.06, 7.03, 7.05-7.06, 8.00-8.01, 9.02-9.04 | MCB | RILs (B73 × CML103) | Samayoa et al. (2015) |
| 2 | 1.02, 1.06, 1.12, 3.05, 8.05, 9.04 | MCB | RILs (EP39 x EP42); (B73 x Mo17) | Ordas et al. (2009, 2010) |
| 3 | 1.05, 1.07-1.08, 3.09, 5.03, 5.05, 6.07, 8.05, 9.03, 10.08 | ECB | F_2:3_ families (D06 × D408) | Bohn et al. (2000) |
| 4 | 1.01-1.02, 1.06, 1.07-1.08, 2.03-2.04, 2.05, 2.07, 2.08, 3.01, 3.04, 3.06, 3.07-3.08, 4.01, 5.04, 5.05, 7.02-7.03, 7.04, 8.03, 8.08, 9.01, 9.03-9.05, 10.04 | ECB | F_2:3_ families & RILs (H99 x Mo17) & (B73 x B52) | Schon et al. (1993); Cardinal et al. (2001, 2006); Cardinal and Lee (2005) |
| 5 | 1.01, 1.02, 1.11, 2.07, 3.02, 3.04, 3.05, 4.01-4.02, 4.03, 5.02, 5.03, 5.07, 6.01, 6.05, 7.01-7.03, 7.05, 8.03, 8.04-8.05, 9.00, 9.08, 10.03, 10.06 | ECB | F_2:3_ families & RILs (B73 x De811) | Krakowsky et al. (2002, 2004) |
| 6 | 1.01, 1.02, 1.07, 2.04, 3.09, 5.03, 5.04, 5.05, 5.07, 6.06-6.07, 7.04, 7.05, 8.04-8.05, 10.04 | ECB | F_2:3_ families and test cross progeny (D06 × D408) | Papst et al. (2004) |
| 7 | 1.01, 1.06, 1.11, 2.01, 2.09, 4.01. 4.06, 5.05, 5.08, 6.00, 6.02, 6.07, 8.03, 8.06, 9.02 | ECB | F_2:3_ families (B73 x Mo47) | Jampatong et al. 2002) |
| 8 | 1.01-1.02, 1.03-1.04, 1.06, 1.07, 1.08, 1.10,1.11, 2.02, 3.05, 3.07-3.09, 4.04, 5.02, 5.04, 5.05-5.06, 5.07, 6.02, 6.04-6.05, 6.06, 7.02-7.03, 7.04-7.05, 8.02, 8.03, 8.05, 8.06-8.08, 9.02, 9.03, 9.04, 9.05-9.06, 10.03-10.06 | SWCB | F_2:3_ families & RILs  (CML139 x Ki3; CML67 x CML131; CML67 x CML204; Mp704 x Mo17; Mp708 x A619) | Bohn et al. (1997); Khairallah et al. (1998); Groh et al. (1998a); Willcox et al. (2002); Brooks et al. (2005, 2007) |
| 9 | 1.01, 1.08, 2.04, 2.05, 4.01, 9.01, 9.02, 10.01 | ACB | F_2:3_ families (Mc37 x Zi330) | Xia et al. (2010) |
| 10 | 1.02, 1.03, 1.05, 1.09. 1.11, 2.02, 2.08, 5.02, 5.04, 5.07, 6.02, 6.07, 7.02, 7.03, 7.04, 8.03, 9.03, 9.05, 9.07, 10.04 | FAM | F_2:3_ families (Mp704 x Mo17; Mp708 x A619) | Brooks et al. (2005, 2007) |
| 11 | 1.02, 1.07, 2.05, 2.09, 3.06-3.07, 4.03, 4.08, 6.00, 5.03, 5.05, 6.05, 7.01-7.02 , 8.06, 9.07, 10.04, 10.06-10.07 | MW | F_2:3_ families (CML290 × Muneng-8128 C0 HC1-18-2-1-1) | García-Lara et al. (2009); Castro Alvarez et al. (2015) |
| 12 | 1.03, 1.06, 1.07, 1.11, 2.02-2.03, 3.05, 5.04, 5.05-5.06, 5.07, 7.02, 7.03, 7.04, 9.02-9.03, 9.04, 9.05-9.06, 10.04 | SCB | F_2:3_ families (CML131 x CML67) | Bohn et al. (1996, 1997); Groh et al. (1998b) |
| 13 | 1.04, 2.02, 2.09, 4.06, 6.01, 6.06, 10.04 | CEW | F_2:3_ families (GE37 x FF8) | Byrne et al. (1998) |

^a^Chromosome bin location of QTL peak of the maize genome. Bins divide the genetic map into 100 approximately equal segments of approximately 20 centiMorgans between two fixed Core Marker. The segments are designated with the chromosome number followed by a two-digit decimal (e.g., 1.00, 1.01, 1.02, etc).

^b^MCB: Meditterean corn borer, ECB: European corn borer, SWCB: South western corn borer, ACB: Asian corn borer, FAM: Fall army worm, MW: Maize weevil, SCB: Sugarcane borer, CEW: Corn ear worm

^c^RILs: Recombinant inbred lines.
