# Supplementary material for: Identification of genomic regions associated with shoot fly resistance in maize and their syntenic relationships in the sorghum genome
Source: PLoS One. 2020 Jun 9;15(6):e0234335. doi: 10.1371/journal.pone.0234335 (PMC7282634; doi:10.1371/journal.pone.0234335)
Supplement: S2 Table — (DOCX) [file pone.0234335.s005.docx]

**S2 Table.** **List of the short-listed putative candidate genes present in detected QTL intervals for shoot fly resistance in maize**

| **QTL^a^** | **Maize Gene ID^b^** | **Description** | **Functional Role** |
| --- | --- | --- | --- |
| *qLL1.1*, *qLA1.1*  (*bnlg1178-bnlg1803*) | Zm00001d027852 | homeobox-leucine zipper protein HAT7 | Meristem growth and defense |
|  | Zm00001d027991 | homeodomain-leucine zipper transcription factor TaHDZipI-1 | Meristem growth and defense |
|  | Zm00001d028115 | inactive TPR repeat-containing thioredoxin TTL3-like | Involved in osmotic and salt stress tolerance. May play a role in the control of meristematic cell size during osmotic stress |
|  | Zm00001d028168 | Glycosyl hydrolase family 10 expressed | Biotic, abiotic stresses and cell wall remodeling |
| *qLI1.3*, *qLW1.3*  (*bnlg1083-umc1073*) | Zm00001d027991 | homeodomain-leucine zipper transcription factor TaHDZipI-1 | Meristem growth and defense |
|  | Zm00001d028115 | inactive TPR repeat-containing thioredoxin TTL3-like | Involved in osmotic and salt stress tolerance. May play a role in the control of meristematic cell size during osmotic stress |
|  | Zm00001d028168 | Glycosyl hydrolase family 10 expressed | Biotic, abiotic stresses and cell wall remodeling |
| *qLG1.4*  (*umc1306-bnlg100*) | Zm00001d029658 | TPA: glycosyl hydrolase family 10 | Biotic, abiotic stresses and cell wall remodeling |
|  | Zm00001d030026 | peroxidase 44-like | involved in lignin biosynthesis based on Northern hybridization and expression tests of the peroxidase promoter genes |
|  | Zm00001d030348 | probable receptor kinase At2g42960 | developmental and defense functions that included gametophyte development, pollen-pistil interactions, shoot apical meristem equilibrium |
|  | Zm00001d031359 | reactive oxygen species modulator 1-like | ROS play in cellular physiology to ascertain their position in the life of the plant |
|  | Zm00001d032298 | probable trehalose-phosphate phosphatase 6 | Removes the phosphate from trehalose 6-phosphate to produce free trehalose. Trehalose accumulation in plant may improve abiotic stress tolerance |
|  | Zm00001d033375 | ubiquitin carboxyl-terminal hydrolase 15-like | jasmonic acid mediated signaling pathway |
|  | Zm00001d033420 | MYB-related transcription | Anthocyanin synthesis |
|  | Zm00001d029560 | RING zinc finger protein-like | rice blast fungus infection |
|  | Zm00001d029568 | cysteine protease | Insect resistance |
|  | Zm00001d030191 | Homeobox-leucine zipper protein HAT22Putative uncharacterized protein | Meristem growth and defense |
|  | Zm00001d033005 | homeobox-leucine zipper protein HAT7 | Meristem growth and defense |
|  | Zm00001d033082 | speckle-type POZ protein | Expressed in Arabidopsis trichomes |
| *qLA2.3*, *qLL2.3* (*umc1884-umc1233*) | Zm00001d005379 | trihelix transcription factor GT-3b-like | play a role in the induction of CAM4 in response to pathogen and salt. |
|  | Zm00001d005028 | NAC domain-containing protein 77 | Biotic and abiotic stress |
|  | Zm00001d005875 | homeobox-leucine zipper protein HAT14 | Meristem growth and defense |
|  | Zm00001d006106 | NAC domain-containing protein 18 | Biotic and abiotic stress |
| *qSV2.2*, *qLSW2.2*  (*bnlg1338-bnlg2248*) | Zm00001d002754 | homeobox-leucine zipper protein ATHB-4 | Meristem growth and defense |
|  | Zm00001d002802 | Leucine-rich repeat receptor protein kinase EXS | Meristem growth and defense |
|  | Zm00001d002065 | cysteine protease 1 | Insect resistance |
|  | Zm00001d002285 | NAC domain-containing protein 21/22 | Biotic and abiotic stress |
|  | Zm00001d002428 | EF hand family protein | Biotic and abiotic stress |
|  | Zm00001d002576 | RING zinc finger protein-like | rice blast fungus infection |
|  | Zm00001d002754 | homeobox-leucine zipper protein ATHB-4 | Meristem growth and defense |
|  | Zm00001d002802 | Leucine-rich repeat receptor protein kinase EXS | Meristem growth and defense |
| *qLSP4.1*, *qSG4.1*  (*bnlg1621b-bnlg1189*) | Zm00001d051799 | homeobox-leucine zipper protein ATHB-4 | Meristem growth and defense |
|  | Zm00001d051879 | nudix hydrolase 4 (LOC100282276), mRNA | Biotic and abiotic stress |
| *qSV6.1*  (*bnlg238-phi077*) | Zm00001d035425 | TPA: ribose-5-phosphate isomerase | cell death, pentose-phosphate shunt, non-oxidative branch, vegetative to reproductive phase transition of meristem |
|  | Zm00001d036036 | glutathione S-transferase | protect the cell from oxidative damage |
|  | Zm00001d035390 | thioredoxin H-type | regulates the redox state of the apoplast and influences plant development and stress responses |
|  | Zm00001d035439 | Homeobox protein knotted-1-like 5 Fragment | Alters Vegetative Development by Decreasing Gibberellin Accumulation |
|  | Zm00001d035445 | gamma-glutamylcysteine synthetase1 | role in cadmium resistance in plant cells |
|  | Zm00001d035683 | subtilisin-chymotrypsin inhibitor CI-1B | response to wounding |
|  | Zm00001d051507 | fatty-acid-binding 2-like | play a pivotal role in plant–microbe interactions |
| *qDH9.1*, *qEC9.1* (*umc1420-umc1258*) | Zm00001d045551 | probable phosphatase 2C 13 | regulators of various signal transduction pathways |
|  | Zm00001d045958 | plant UBX domain-containing 8-like | UBX domain-containing protein regulates plant growth |
|  | Zm00001d046039 | target of Myb 1 | play roles in a number of developmental and stress-responsive: *Zea mays* C1 involved in anthocyanin biosynthesis by encoding c-myb-like transcription factor |
|  | Zm00001d046344 | Extracellular sulfatase Sulf-1 | regulate growth factor signaling. |
|  | Zm00001d046625 | TPA: glycosyl hydrolase family 10 | Biotic, abiotic stresses and cell wall remodeling |
|  | Zm00001d046938 | leucine-rich repeat kinase family | structure, function, and signal transduction pathways |
|  | Zm00001d047015 | TPA: cytochrome P450 superfamily | these proteins are important for the biosynthesis of several compounds such as hormones, defensive compounds and fatty acids |
| *qSG9.2*  (*umc1586-bnlg1012*) | Zm00001d047981 | glutamate decarboxylase 1-like | plays a major role in GABA synthesis in plants under normal growth conditions and in response to stress |
|  | Zm00001d048112 | RING zinc finger protein-like | rice blast fungus infection |
|  | Zm00001d047102 | cys2/His2 zinc-finger transcription factor | especially drought stress |
|  | Zm00001d047124 | proline oxidase | abiotic stresses associated with water deprivation |
|  | Zm00001d047220 | serine/threonine-protein kinase SAPK1 | plant-specific serine/threonine kinases involved in plant response to abiotic stresses and abscisic acid (ABA)-dependent plant development |
|  | Zm00001d048047 | probable cyclic nucleotide-gated ion channel 6 | development and as a ‘guard’ in defense against biotic and abiotic challenges |
| *qSG9.3*  (*umc1078-umc1657*) | Zm00001d047601 | SNF1-related protein kinase regulatory subunit beta-1 | allow plants to tolerate herbivory by allocating carbon to roots |
|  | Zm00001d047754 | fatty acid desaturase7 | enhances plant defenses against aphids |
|  | Zm00001d048007 | Histone H2A | growth and development, phase transitions, and response to the environment |
|  | Zm00001d048009 | GTP-binding protein | Plant defense responses at stomata and apoplast are the most important early events during plant-bacteria interactions |
|  | Zm00001d048009 | 1-O-acylceramide synthase | powerful defensive mechanism against pathogens |

^a^Putative QTL are designated by the corresponding chromosome in which they are found

^b^Gene model set Zm00001d.2 corresponds to Gramene release 36

LL: leaf length, LW: leaf width, LA:leaf area, LI: leaf injury, LSW: leaf surface wetness, LSP: leaf sheath pigmentation, LG: leaf glossiness, SV: seedling vigor, SG: stem girth, EC: oviposition, DH: deadheart
